# Supplementary material for: Predictors and outcomes of peritoneal dialysis-related infections due to filamentous molds (MycoPDICS)
Source: PLoS One. 2022 May 24;17(5):e0268823. doi: 10.1371/journal.pone.0268823 (PMC9129032; doi:10.1371/journal.pone.0268823)
Supplement: S1 Fig — (DOCX) [file pone.0268823.s001.docx]

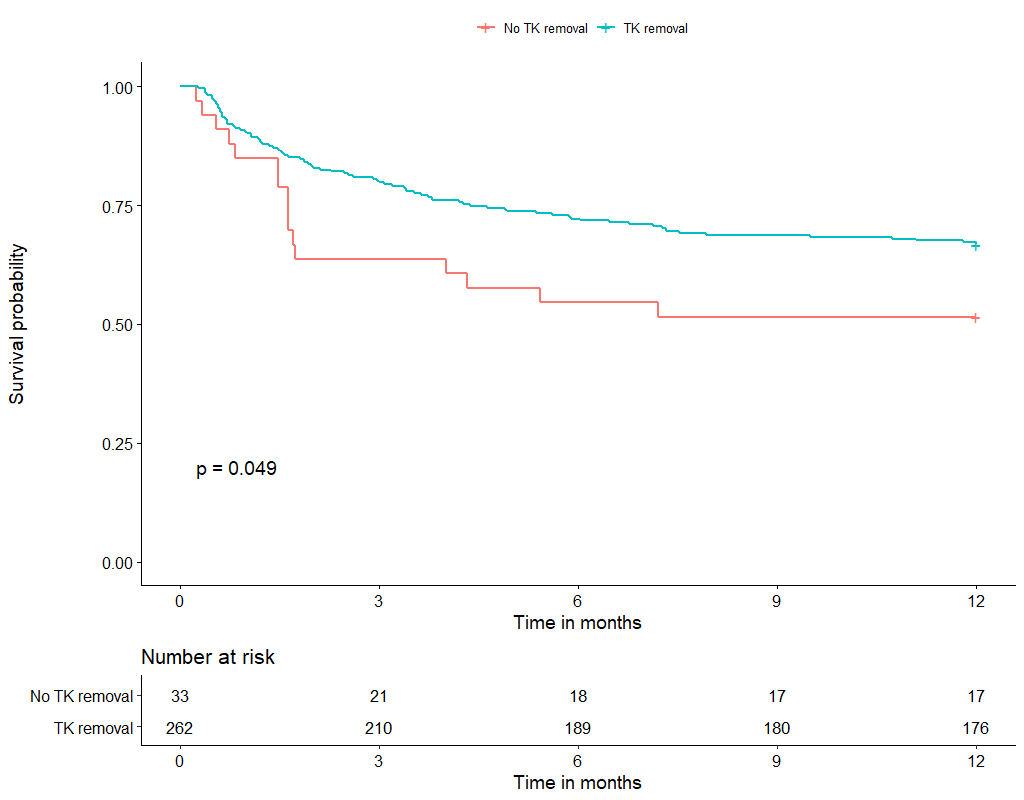


**Supplemental Figure 1.** Kaplan-Meier curves comparing patient survival following PD-related fungal peritonitis treated with and without PD catheter removal.
