# Supplementary material for: Predictors and outcomes of peritoneal dialysis-related infections due to filamentous molds (MycoPDICS)
Source: PLoS One. 2022 May 24;17(5):e0268823. doi: 10.1371/journal.pone.0268823 (PMC9129032; doi:10.1371/journal.pone.0268823)
Supplement: S1 Table — (DOCX) [file pone.0268823.s002.docx]

**S1 Table.** Sensitivity analyses of duration of antifungal therapies and mortality among patients with fungal peritonitis using univariable and multivariate analyses.

| **Variables** | **Unadjusted HR** | **95% CI** | ***P* value** | **Adjusted HR model 1^a^** | **95% CI** | ***P* value** | **Adjusted HR model 2^b^** | **95% CI** | ***P* value** |
| --- | --- | --- | --- | --- | --- | --- | --- | --- | --- |
| **Among patients with adequate duration of treatment (after TK removal)** | | | | | | | | | |
| *Duration of antifungal treatment per 1-week increment from 14 days of treatment after TK removal* | 1.18 | 1.05–1.34 | 0.008 | 1.13 | 0.99–1.30 | 0.07 | 1.11 | 0.96–1.28 | 0.16 |
| *Received treatment 15-21 days after TK removal (Reference 14 days after TK removal)* | 1.92 | 0.84–4.40 | 0.12 | 2.01 | 0.82–4.92 | 0.13 | 1.94 | 0.75–5.02 | 0.17 |
| *Received treatment 22-28 days after TK removal* | 1.56 | 0.57–4.29 | 0.39 | 1.53 | 0.47–4.93 | 0.48 | 1.66 | 0.49–5.59 | 0.41 |
| *Received treatment 29 days or more after TK removal* | 2.57 | 1.12–5.87 | 0.03 | 1.79 | 0.68–4.76 | 0.24 | 1.92 | 0.65–5.68 | 0.24 |
| **Among patients with at least 2 weeks of treatment** | | | | | | | | | |
| *Duration of antifungal treatment per 1-week increment from 14 days of treatment* | 1.16 | 1.04–1.30 | 0.008 | 1.12 | 0.98–1.27 | 0.09 | 1.11 | 0.97–1.26 | 0.12 |
| *Received treatment 15-21 days (Reference 14 days treatment)* | 2.15 | 1.11–4.13 | 0.02 | 1.95 | 0.87–4.40 | 0.11 | 1.98 | 0.82–4.75 | 0.13 |
| *Received treatment 22-28 days* | 1.42 | 0.54–3.71 | 0.47 | 1.10 | 0.36–3.35 | 0.86 | 1.12 | 0.36–3.47 | 0.85 |
| *Received treatment 29 days or more* | 2.32 | 1.14–4.72 | 0.02 | 1.86 | 0.81–4.31 | 0.15 | 1.97 | 0.81–4.79 | 0.13 |
| **Among patients with antifungal treatment** | | | | | | | | | |
| *Combination of antifungal* | 0.73 | 0.39–1.38 | 0.34 | 0.54 | 0.24–1.19 | 0.12 | 0.58 | 0.26–1.30 | 0.18 |

**Abbreviations:** CI, confidence interval; FP, fungal peritonitis; HR, hazard ratio; PDE, peritoneal dialysis effluent; TK, Tenckhoff catheter

^a^Adjusted for age, gender, diabetes, employed state, PD vintage, hemoglobin, serum albumin, PDE leukocyte count > 1,090 cells/µL.

^b^Adjusted for age, gender, diabetes, employed state, PD vintage, hemoglobin, serum albumin, PDE leukocyte count > 1,090 cells/µL and fungal characteristic.
