# Supplementary material for: Predictors and outcomes of peritoneal dialysis-related infections due to filamentous molds (MycoPDICS)
Source: PLoS One. 2022 May 24;17(5):e0268823. doi: 10.1371/journal.pone.0268823 (PMC9129032; doi:10.1371/journal.pone.0268823)
Supplement: S1 File — (DOCX) [file pone.0268823.s003.docx]

**Predictors and Outcomes of Peritoneal Dialysis (PD)-Related Infections due to Filamentous Molds**

**PROJECT LEADER**

Professor Talerngsak Kanjanabuch, M.D.

Division of Nephrology, Department of Medicine, Faculty of Medicine, Chulalongkorn University, Thailand

Center of Excellence in Kidney Metabolic Disorders, Faculty of Medicine, Chulalongkorn University, Thailand

Peritoneal Dialysis Excellent Center, King Chulalongkorn Memorial Hospital, Bangkok, Thailand

**PROJECT TEAM**

- Tanawin Nopsopon, M.D.

Department of Preventive and Social Medicine, Faculty of Medicine, Chulalongkorn University, Thailand

- Associate Professor Krit Pongpirul, M.D.

Department of Preventive and Social Medicine, Faculty of Medicine, Chulalongkorn University, Thailand

- Professor Somchai EIam-Ong, M.D.

Division of Nephrology, Department of Medicine, Faculty of Medicine, Chulalongkorn University, Thailand

**INTRODUCTION**

Not only have fungal peritonitis in patients with peritoneal dialysis (PD) markedly increases incidence compare to the general population, but also causing high mortality ranging 4.0-60.5%. Retrospective studies and registry demonstrated that prevalence of fungal peritonitis varies from approximately 1% to 15% of all peritonitis episodes in people undergoing PD, episode ranging from 0.02 episode/patient-year. A higher prevalence was reported from topical countries. Although about 80 fungi species have been demonstrated as potentially causative, *Candida* spp. is predominant pathogen causing fungal peritonitis. The ratio of prevalence of *Candida*- over non-candida-peritonitis was roughly 3:1-9:1. Recently, International Society for Peritoneal Dialysis (ISPD) 2016 mandates to remove the PD catheter immediately after fungi are identified in PD patients with fungal peritonitis (1C), followed by continuation of anti-fungal agents for an additional 2 more weeks (2C). However, the guideline has given no specific recommendation on type and dose of anti-fungal medications (ref). Moreover, this recommendation is based on studying in *Candida* peritonitis. Until now, only 3 retrospective studies compare the peritonitis outcome among different causative fungal species. Wong, et al. demonstrated an equal mortality rate between Candida and non-Candida species (47% vs. 37%). However, Lo et al. have shown that *C. parapsilosis* is an independent risk factor for mortality. In Korea study, *C. albicans* infection is independent predictors for mortality in the univariate analysis but loss its prediction in the multivariate analysis. However, the results of previous studies should be interpreted because of a very small number of filamentous mold peritonitis episodes in each group. The number of filamentous mold in most series is less than 25 cases; the highest number was 21 from Hong Kong (Wong study). Therefore predictors, manifestations, and outcomes and effect of antifungal selection on patient mortality were compared between peritonitis caused by mold and yeast.

**REVIEW LITERATURES**

Fungal peritonitis is a rare but serious complication of peritoneal dialysis (PD) and is associated with significant mortality. Observational studies suggest that fungal peritonitis accounts for approximately 3% to 15% of all peritonitis episodes in people undergoing PD. Clinical signs and symptoms of fungal peritonitis are non-specific and can resemble bacterial peritonitis. On presentation, it is common for there to be few signs of peritonitis; although catheter obstruction [1] caused by fungal casts is an exception. The Gram stain may be positive but a high degree of suspicion must be maintained for cultures and Gram stains that are negative. The type of fungus determines the success rate of return to PD. Rates of successful return to PD appear to be reduced after filamentous fungal peritonitis. Although about 80 fungi species and other organisms have been reported as potentially infective, Candida spp. are the most common fungi associated with fungal peritonitis. Fungal colonisation of the PD catheter may be visible. Reported mortality rates vary. An overall mortality rate of 60.5% has been reported but this rate escalated to 94.4% for patients whose catheters were in situ at one month [9]. It was further reported that two-thirds (66.6%) of patients whose catheter removal was delayed (median duration 7 days) died and the mortality rate at one month among patients whose catheters were removed at less than 24 hours was 18.8%. [9] Other studies have also documented that mortality rates rise when PD catheter removal is delayed.

The optimal choice of antifungals has not been established. Until recently, interpretating treatment data has been difficult due to the lack of standardised sensitivity testing. The degree of standardisation of testing across centres and countries remains uncertain. In addition, some antifungals penetrate poorly into the peritoneal cavity and should be avoided if treating fungal peritonitis (e.g. ketoconazole) [47]. Itraconazole also has poor peritoneal penetration. As noted in the ISPD guidelines, intraperitoneal amphotericin should be avoided because it causes pain and increased fibrosis. [48] Specific advice should be obtained from the local infectious disease team or microbiologists.

**REFERENCES**1. Nankivell BJ, Pacey D, Gordon DL. Peritoneal eosinophilia associated with Paecilomyces variotii infection in continuous ambulatory PD. Am J Kidney Dis 1991; 18(5):603-05.

2. Bibashi E, Papagianni A, Kelesidis A, Antoniadou R, Papadimitriou M. Peritonitis due to Aspergillus niger in a patient on continuous ambulatory PD shortly after kidney graft rejection. Nephrol Dial Transplant 1993; 8(2): 185-87.

3. Alscher DM, Pfinder-Nohe E, Rumpf D, Pauli-Magnus C, Knabbe C, Kuhlmann U, et al. Containers for waste causing peritonitis in CAPD patients. EDTNA-ERCA J 2001; 27(3): 140- 42.

4. Eisenberg ES, Alpert BE, Weiss RA, Mittman N, Soeiro R. Rhodotorula rubra peritonitis in patients undergoing continuous ambulatory PD. Am J Med 1983; 75(2): 349-52.

5. O'Connor JP, Nimmo GR, Rigby RJ, Petrie JJ, Hardie IR, Strong RW. Algal peritonitis complicating continuous ambulatory PD. Am J Kidney Dis 1986; 8(2): 122-23.

6. Bordes A, Campos-Herrero MI, Fernadez A, Vega N, Rodriguez JC, Palop L. Predisposing and prognostic factors of fungal peritonitis in PD. Perit Dial Int 1995; 15: 275-76.

7. Chan TM, Chan CY, Cheng SW, Lo WK, Lo CY, Cheng IK. Treatment of fungal peritonitis complicating continuous ambulatory PD with oral fluconazole: a series of 21 patients. Nephrol Dial Transplant 1994; 9(5): 539-42.

8. Chen CM, Ho MW, Yu WL, Wang JH. Fungal peritonitis in PD patients: effect of fluconazole treatment and use of the twin-bag disconnect system. J Microbiol Immunol Infect 2004; 37(2): 115-20.

9. Ram R, Swarnalatha G, Neela P, Murty KV. Fungal peritonitis in patients on continuous ambulatory PD: a single-centre experience in India. Nephron Clin Pract 2008; 110(4): c207- 12.

10. Lo WK, Chan CY, Cheng SW, Poon JF, Chan DT, Cheng IK. A prospective randomized control study of oral Nystatin prophylaxis for Candida peritonitis complicating continuous PD. American Journal of Kidney Diseases. 1996; 28(4): 549-552.

11. Ampel NM, White JD, Varanasi UR, Larwood TR, Van Wyck DB, Galgiani JN. Coccidioidal peritonitis associated with continuous ambulatory PD. Am J Kidney Dis 1988; 11(6): 512-14.

12. Smith JW, Arnold WC. Cryptococcal peritonitis associated with continuous ambulatory PD. Am J Kidney Dis 1988; 11: 430-33.

13. Ram R, Swarnalatha G, Varma V, Desai M, Prasad N, Sastry RA, et al. Pseudoaneurysm of left colic artery after Tenckhoff catheter removal, with unrecognized fungal peritonitis. J Nephrol 2008; 21(6): 962-64.

14. Lo WK, Chan CY, Cheng SW, Poon JF, Chan DT, Cheng IK. A prospective randomized control study of oral nystatin prophylaxis for Candida peritonitis complicating continuous ambulatory peritoneal dialysis. Am J Kidney Dis 1996; 28(4): 549-52.

15. Restrepo C, Chacon J, Manjarres G. Fungal peritonitis in peritoneal dialysis patients: successful prophylaxis with fluconazole, as demonstrated by prospective randomized control trial. Perit Dial Int 2010; 30: 619-25.

16. Prabhu MV, Subhramanyam SV, Gandhe S, Antony SK, Nayak KS. Prophylaxis against fungal peritonitis in CAPD – a single center experience with low-dose fluconazole. Ren Fail 2010; 32: 802-05.

17. Moreiras-Plaza M, Vello-Román A, Sampróm-Rodríguez M, Feijóo-Piñeiro D. Ten years without fungal peritonitis: a single center’s experience. Perit Dial Int 2007; 27 (4): 460-63.

18. Wong PN, Lo KY, Tong GM, Chan SF, Lo MW, Mak SK, et al. Prevention of fungal peritonitis with nystatin prophylaxis in patients receiving CAPD. Perit Dial Int 2007; 27: 531-36.The KHA-CARI Guidelines – Caring for Australasians with Renal Impairment Peritonitis Treatment and Prophylaxis (February 2014) Page 18

19. Williams PF, Moncrieff N, Marriott J. No benefit in using nystatin prophylaxis against fungal peritonitis in PD patients [letter]. Perit Dial Int 2000; 20(3): 352-53.

20. Thodis E, Vas SI, Bargman JM, Singhal M, Chu M, Oreopoulos DG. Nystatin prophylaxis: its inability to prevent fungal peritonitis in patients on continuous ambulatory peritoneal dialysis. Perit Dial Int 1998; 18: 583-89.

21. Wadhwa NK, Suh H, Cabralda T. Antifungal prophylaxis for secondary fungal peritonitis in PD patients: Adv Perit Dial 1996; 12: 189-91. 22. Robitaille P, Merouani A, Clermont M-J, Hebert E. Successful antifungal prophylaxis in chronic peritoneal dialysis: a pediatric experience. Perit Dial Int 1995; 15: 77-79.

23. Zaruba K, Peters J, Jungbluth H. Successful prophylaxis for fungal peritonitis in patients on continuous ambulatory PD: six years’ experience. Am J Kidney Dis 1991; 17(1): 43-46.

24. Charlier C, Hart E, Lefort A, Ribaud P, Dromer F, Denning DW, et al. Fluconazole for the management of invasive candidiasis: where do we stand after 15 years? J Antimicrob Chemother 2006; 57(3): 384-410.

25. Li PKT, Leung CB, Leung AKL, Luk WK, Lai KN. Posthysteroscopy fungal peritonitis in a patient on continuous ambulatory peritoneal dialysis. Am J Kidney Dis 1993; 21(4): 446-48.

26. Maruyama H, Nakamaru T, Oya M, Miyakawa Y, Sato N, Ishizuka Y, et al. Posthysteroscopy Candida glabrata peritonitis in a patient on CAPD. Perit Dial Int 1997; 17(4): 404-05.

27. McQuillan RF, Chiu E, Nessin S, Lok CE, Roscoe JM, Tam P, et al. A randomized controlled trial comparing mupirocin and polysporin triple ointments in peritoneal dialysis patients: the MP3 Study. Clin J Am Soc Nephrol 2012; 7(2): 297-303.

28. Miles R, Hawley CM, McDonald SP, Brown FG, Rosman JB, Wiggins KJ, et al. Predictors and outcomes of fungal peritonitis in peritoneal dialysis patients. Kidney Int 2009; 76(6): 622- 28.

29. Wang AYM, Yu AWY, Tao PK, Lam PKW, Leung CB, Lai KN, et al. Factors predicting outcome of fungal peritonitis: analysis of a 9-year experience of fungal peritonitis in a single centre. Am J Kidney Dis 2000; 36(6): 1183-92.

30. Chen KH, Chang CT, Yu CC, Huang JY, Yang CW, Hung CC. Candida parapsilosis peritonitis has more complications than other Candida peritonitis in peritoneal dialysis patients. Ren Fail 2006; 28(3): 241-46.

31. Raaijmakers R, Schröder C, Monnens L, Cornelissen E, Warris A. Fungal peritonitis in children on peritoneal dialysis. Pediatr Nephrol 2007; 22: 288-93.

32. Levallois J, Nadeau-Fredette A-C, Labbé A-C, Laverdière M, Ouimet D, Valée M. Ten-year experience with fungal peritonitis in peritoneal dialysis patients: antifungal susceptibility patterns in a North American center. Int J Infect Dis 2012; 16(1): e41-43.

33. Khan FH, Elsayed M, Anand D, Khattab MA, Sanjay D. Fungal peritonitis in patients undergoing continuous ambulatory peritoneal dialysis in Qatar. J Infect Dev Ctries 2011; 5(9): 646-51.

34. Ghali JR, Bannister KM, Brown FG, Rosman JB, Wiggins KJ, Johnson DW, et al. Microbiology and outcomes of peritonitis in Australian peritoneal dialysis patients. Perit Dial Int 2011; 31(6): 651-62.

35. Mujais S. Microbiology and outcomes of peritonitis in North America. Kidney Int Suppl 2006; 70: S55-S62.

36. Prasad KN, Prasad N, Gupta A, Sharma RK, Verma AK, Ayyagari A. Fungal peritonitis in patients on continuous ambulatory peritoneal dialysis: single centre Indian experience. J Infect 2004; 48(1): 96-101. The KHA-CARI Guidelines – Caring for Australasians with Renal Impairment Peritonitis Treatment and Prophylaxis (February 2014) Page 19

37. Chang TI, Kim HW, Park JT, Lee DH, Lee JH, Yoo TH, et al. Early catheter removal improves patient survival in peritoneal dialysis patients with fungal peritonitis: results of ninety-four episodes of fungal peritonitis at a single center. Perit Dial Int 2011; 31(1): 60-66.

38. DeVault GA Jr, Brown ST 3rd, King JW, Fowler M, Oberle A. Tenckhoff catheter obstruction resulting from invasion by Curvularia lunata in the absence of peritonitis. Am J Kidney Dis 1985; 6(2): 124-27.

39. McNeeley DJ, Vas SI, Dombros N, Orepoulos DG. Fusarium peritonitis: an uncommon complication of continuous ambulatory peritoneal dialysis. Perit Dial Int 1981; 1(6): 94-96.

40. Kerr CM, Perfect JR, Craven PC, Jorgensen JH, Drutz DJ, Shelburne JD, et al. Fungal peritonitis in patients on continuous ambulatory peritoneal dialysis. Ann Intern Med 1983; 99(3): 334-36.

41. Crompton CH, Balfe JW, Summerbell RC, Silver MM. Peritonitis with Paecilomyces complicating peritoneal dialysis. Pediatr Infect Dis J 1991; 10(11): 869-71.

42. Huang JW, Chu TS, Wu MS, Peng YS, Hsieh BS. Penicillium spp. colonization plaques on a Tenckhoff catheter without resultant peritonitis in a peritoneal dialysis patient. Nephrol Dial Transplant 2000; 15(11): 1872-73.

43. Park SB, Kim KH, Joo I, Kim HC. Scanning electron microscopy studies of peritoneal catheter in CAPD peritonitis due to Aspergillus fumigatus. Perit Dial Int 1996; 16(1): 81-83.

44. Sekkarie M, Holmes C, Ranjit U, Kauffman CA. Grossly visible fungal colonization of a Tenckhoff catheter. A case report and literature review. Perit Dial Int 1991; 11(1): 85-87.

45. Vargemezis V, Passadakis P, Liakopoulos V, Kriki P, Kantartzi A. Fungal colonization of peritoneal catheter with persistently sterile cloudy effluent in the absence of clinical findings of fungal peritonitis. Perit Dial Int 2004; 24(1): 81-84.

46. Snyder S. Peritonitis due to Saccharomyces cerevisiae in a patient on CAPD. Perit Dial Int 1992; 12(1): 77-78.

47. Lee SH, Chiang SS, Hseih SJ, Shen HM. Successful treatment of fungal peritonitis with intracatheter antifungal retention. Adv Perit Dial 1995; 11: 172-75.

48. Chapman JR, Warnock DW. Ketoconazole and fungal CAPD peritonitis. Lancet 1983; 2(8348): 510-11.

49. Piraino B, Bailie GR, Bernardini J, Boeschoten E, Gupta A, Holmes C, et al. Peritoneal dialysis-related infections recommendations: 2005 update. Perit Dial Int 2005; 25: 107-31.

50. Gilbert DN, Moellering RC, Jr, Eliopoulos GM, editors. The Sanford Guide to Antimicrobial Therapy. 2012, 42nd edition. Sperryville, Va: Antimicrobial Therapy Inc. USA; 2012.

51. Madariaga MG, Tenorio A, Proia L. Trichosporon peritonitis treated with caspofungin. J Clin Microbiol 2003; 41(12): 5827-29.

52. Fourtounas C, Marangos M, Kalliakmani P, Savidaki E, Goumenos DS, Vlachojannis JG. Treatment of peritoneal dialysis-related fungal peritonitis with caspofungin plus amphotericin B combination therapy. Nephrol Dial Transplant 2006; 21(1): 236-37.

53. Chang BP-H, Sun P-L, Huang F-Y, Tsai T-S, Lin C-C, Lee M-D, et al. Paecilomyces lilacinus peritonitis complicating peritoneal dialysis cured by oral voriconazole and terbinafine combination therapy. J Med Microbiol 2008; 57: 1581-84.

54. Ghebremedhin B, Bluemel A, Neumann K-H, Koenig B, Koenig W. Peritonitis due to Neosartorya pseudofischeri in an elderly patient undergoing peritoneal dialysis successfully treated using voriconazole. J Med Microbiol 2009; 58: 678-82.

55. Pimental JD, Dreyer G, Lum GD. Peritonitis due to Cunninghamella bertholletiae in a patient undergoing continuous ambulatory peritoneal dialysis. J Med Microbiol 2006; 55: 115-18.The KHA-CARI Guidelines – Caring for Australasians with Renal Impairment Peritonitis Treatment and Prophylaxis (February 2014) Page 20

56. Sedlacek M, Cotter JG, Suriwinta AA, Kaneko TM, Zuckerman RA, Parsonnet J, et al. Mucormycosis peritonitis: more than 2 years of disease-free follow up after posaconazole salvage therapy after failure of liposomal amphotericin B. Am J Kidney Dis 2008; 51(2): 302- 06.

57. Verghese S, Palani R, Thirunavakarasu N, Chellamma T, Pathipata P. Peritonitis due to Aspergillus terreus in a patient undergoing continuous ambulatory peritoneal dialysis. Mycoses 2008; 51(2): 174-76.

58. Johnson RJ, Ramsey PG, Gallagher N, Ahmad S. Fungal peritonitis in patients on PD: incidence, clinical features and prognosis. Am J Nephrol 1985; 5(3): 169-75.

59. Warady BA, Bashir M, Donaldson LA. Fungal peritonitis in children receiving PD: a report of the NAPRTCS. Kidney Int 2000; 58(1): 384-89.

60. Von Schnakenburg C, Feneberg R, Plank C, Zimmering M, Arbeiter K, Bald M, et al. Percutaneous gastrostomy in children on peritoneal dialysis. Perit Dial Int 2006; 26: 69-77.

61. Li PK-T, Szeto CC, Piraino B, Bernardini J, Figueiredo AE, Gupta A, et al. Peritoneal dialysisrelated infections recommendations: 2010 update. ISPD guidelines/recommendations. Perit Dial Int 2010; 30: 393–423.

62. Piraino B, Bernardini J, Brown E, Figueiredo A, Johnson DW, Lye W-C, et al. ISPD position statement on reducing the risks of peritoneal dialysis-related infections. Perit Dial Int 2011; 31(6): 614-30

**OBJECTIVES
*Primary objective***

- To assess predictors, manifestations, and outcomes and effect of antifungal selection on patient mortality were compared between peritonitis caused by mold and yeast.

***Secondary objectives***

- To assess predictors, manifestations, and outcomes and effect of antifungal selection on patient mortality were compared between fungal peritonitis with and without fulfilling peritonitis criteria.
- To assess predictors of treatment responsiveness in patients with fungal peritonitis

**SCOPE OF THE STUDY**

A retrospective study includes adult PD patients at age of above 18 years old from the Thailand Fungal PD-related Infectious Complications Surveillance Data (MycoPDICS DATA) who have fungal peritonitis and catheter-related fungal infection during July 2015-June 2020.

**METHADOLOGY**

***Study design and population***

This retrospective study will include adult PD patients at age of above 18 years old from the Thailand Fungal PD-related Infectious Complications Surveillance Data (MycoPDICS DATA) who have fungal peritonitis and catheter-related fungal infection during July 2015-June 2020. To be eligible for inclusion, the patients have to fulfill the diagnosis criteria of the ISPD guideline 2016 by the presence of at least 2 of the following: 1) presence of clinical symptoms of peritoneal inflammation, including abdominal pain and cloudy dialysate; 2) presence of more than 100 leukocytes/mm^3^ dialysate with at least 50% neutrophils; 3) documentation of fungi in dialysate or PD catheter by either smear or culture (ref).

***Data collection***

The MycoPDIC Data is a national registry designed to survey the incidence of PD-related infections with fungus or environmental organisms under the umbrella of the Nephrology Society of Thailand (NST). The objectives of this surveillance include 1) monitoring fungal and environmental infection in PD patients, disease trends and risk factors, 2) estimating the burden of disease and detecting the first clusters of an outbreak, 3) providing pathogen isolates for the outbreak investigation, detecting new pathogen, and monitoring an emerging antifungal resistance, and 4) evaluating intervention and supporting treatment platform. The NST undertakes a verification procedure through communication with the treating physicians and reference PD nurses to confirm the index case. Single case investigation involves 1) communication with the index patient and/or their relatives using a semi-structured questionnaire on possible risk factors for infection and picturing house-hold environment 2) transport the suspected PD specimens to the central laboratory within 24 hours in an ice-shield container for organism identification, including PD bag, serum, and PD catheters before and after (as needed) antifungal treatment. Identified species and anti-fungal agent susceptibility summaries are distributed back to the reporting units as soon as an availability of the results. This surveillance collected patient-level and facility-level data using a standard protocol and data collection instruments in all voluntary participating facilities. The information, including patient’s medical record, potential risk factors, the initial and subsequent treatment, and laboratory data is retrieved and submitted to NST by a paper-based system, with manual completion of the case-record form and manual data entry.

The retrieved data for this study included patient demographics, comorbidities at the start of dialysis, presenting symptoms and signs, presence of coexisting bacterial peritonitis, antibiotic use within the 3 months prior to fungal peritonitis, laboratory data at the onset of peritonitis, species of pathologic fungus and fungal characteristics, the initial and subsequent antimicrobial treatment, catheter removal and the time of removal, and patient outcomes.

***Definition***

PD catheter malfunction defines as mechanical failure in dialysate inflow or outflow. Death related to fungal peritonitis is defined as death of a patient with active peritonitis or sepsis secondary to peritonitis or within 4 weeks of diagnosis of fungal peritonitis (ref). Fungi will categorized into 4 categories according to wet smear, including yeast, hyaline mold, non-hyaline mold, and multiple fungal organisms. In case of polymicrobial infection, fungal peritonitis is included if a fungus was at least one of isolated organism.

***Statistical analysis***

Results will be expressed as frequencies and percentages for categorical variables and median and IQR for continuous variables. Differences between two groups of patients will be analyzed by χ2-test and Fisher's exact test for categorical data, and Mann–Whitney U test for continuous data. All major patient outcomes will be analyzed by survival analyses using Kaplan Meier curves together with the Log-rank test. Associations between variables and patient outcomes were first analyzed by univariate Cox proportional hazard regression. All variables with P value of 0.20 or less were candidate for multivariable Cox model with adjustment for age, gender, diabetes, employed state, PD vintage, hemoglobin, serum albumin, and high PDE leukocyte count. The assumption of proportional hazard was verified using Schoenfeld residuals and plots. The final model was adjusted for age, gender, diabetes, employed state, PD vintage, hemoglobin, serum albumin, high PDE leukocyte count, wet smear fungal characteristics, PD catheter removal, and duration of antifungal therapy. Data were analyzed using the software packages Stata 16.1 (College Station, TX) and R 4.0.5 (R Core Team, Vienna). P-values less than 0.05 were considered statistically significant.

**DURATION OF PROJECT**

3 months

**PROJECT PLAN**

| **ACTIVITIES** | **M1** | **M2** | **M3** | **OUTPUT** |
| --- | --- | --- | --- | --- |
| **Preparation of work** | **√** |  |  |  |
| **Data collection** | **√** | **√** |  |  |
| **Data analysis** |  | **√** | **√** |  |
| **Data report and publication** |  |  | **√** | ≥2 publications |

**BUDGETS**

| **LISTS** | **AMOUNTS (BAHT)** |
| --- | --- |
| - General Operating Expenses | 15,000 |
| - Service-Related Expenses | 10,000 |
| - Other (Publication fee) | 50,000 |
| **TOTAL** | 75,000 |

**SIGNATURE OF PROJECT LEADER:**

**(PROFESSOR TALERNGSAK KANJANABUCH, M.D.)**
